# Application of “8”-loop traction-assisted duodenal endoscopic submucosal dissection and defect closure

**DOI:** 10.1055/a-2590-8339

**Published:** 2025-05-22

**Authors:** Qianqian Chen, Shuai Tian, Kunming Lv, Enqiang Linghu

**Affiliations:** 1 104607 Department of Gastroenterology, The First Medical Center, Chinese PLA General Hospital, Beijing, China; 2 104607 Department of Gastroenterology, 970 Hospital of the PLA Joint Logistic Support Force, Yantai, China

Duodenal endoscopic submucosal dissection (D-ESD) and duodenal defect endoscopic closure remain technically challenging due to the organ’s anatomical complexity, including its narrow lumen, acute angulation, and thin wall, which increases the risk of perforation and bleeding 1 . The duodenum’s proximity to critical structures, such as the pancreas and bile ducts, further complicates endoscopic interventions, making it a high-risk area for endoscopic resection 2 .

Traditional ESD techniques often struggle with inadequate traction and poor visualization, which can lead to incomplete resection or unintended tissue damage. To address these challenges, various traction methods have been developed, including clip-and-line traction, magnetic anchor guidance, and rubber band traction 3 . Among these, the “8”-loop traction technique has emerged as a promising approach, providing continuous and adjustable traction during dissection, thereby improving visualization and procedural stability 4 . This case report describes the successful application of the “8”-loop traction-assisted ESD technique combined with the defect closure in a patient with early duodenal cancer (EDC).

A 65-year-old man was admitted to our hospital with a 2.5 × 2.0-cm EDC on the intestinal wall opposite to the duodenal papilla. We resected the lesion using super minimally invasive surgery, also known as ESD ( Fig. 1 , Video 1 ). The mucosal layer around the lesion was injected and circumferentially incised. An “8”-loop traction device was applied, with one end fixed to the lesion and the other end anchored to the opposite intestinal wall. This traction method provided clear visualization by separating the submucosal layer from the muscularis propria. The lesion of EDC was finally completely resected and left a large defect of approximately 4.0 × 3.5 cm. After achieving hemostasis, the “8”-loop was first fixed to the oral side of the defect and the opposite side with tradition clips. Then, clips were used to clamp the wound edge pulled closely by “8”-loop until the defect was completely closed.

**Fig. 1:**
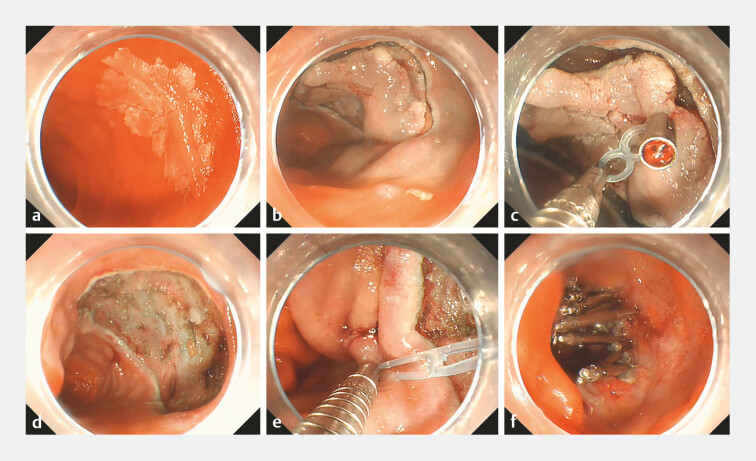
“8”-loop traction-assisted ESD and defect closure for duodenal tumor. **a** a 2.5 × 2.0-cm early duodenal cancer on the intestinal wall opposite to the duodenal papilla. **b** Circumferentially incision of the duodenal lesion. **c** An “8”-loop traction device was applied to assist ESD. **d** A large defect of approximately 4.0 × 3.5 cm. **e** An “8”-loop traction device was applied to assist defect closure. **f** The defect was completely closed.

“8”-loop traction-assisted ESD and defect closure for duodenal tumor.Video 1

The combination of “8”-loop traction-assisted ESD and defect closure techniques represents a promising approach for the resection of duodenal tumors. And this technique improves procedural safety and efficacy, particularly in challenging anatomical locations.

Endoscopy_UCTN_Code_TTT_1AO_2AG_3AD

## Notes

Endoscopy E-Videos https://eref.thieme.de/e-videos
*E-Videos* is an open access online section of the journal *Endoscopy* , reporting on interesting cases and new techniques in gastroenterological endoscopy. All papers include a high-quality video and are published with a Creative Commons CC-BY license. Endoscopy E-Videos qualify for HINARI discounts and waivers and eligibility is automatically checked during the submission process. We grant 100% waivers to articles whose corresponding authors are based in Group A countries and 50% waivers to those who are based in Group B countries as classified by Research4Life (see: https://www.research4life.org/access/eligibility/ ). This section has its own submission website at https://mc.manuscriptcentral.com/e-videos .

## References

[1] Ma M Liu S Wang J Closure of a large post-endoscopic submucosal dissection mucosal defect in the duodenum with a novel through-the-scope twin clip Endoscopy 2023 55 E523 E524 10.1055/a-2024-9901 36894150 PMC9998218

[2] Shi L Long F Xu H Chronic esophagotracheal fistula secondary to esophageal diverticulum successfully treated by endoscopic submucosal dissection and dual action tissue clip Endoscopy 2023 55 E1128 E1130 10.1055/a-2163-2050 37875154 PMC10597680

[3] Mohammed A Gonzaga ER Hasan MK Low delayed bleeding and high complete closure rate of mucosal defects with the novel through-the-scope dual-action tissue clip after endoscopic resection of large nonpedunculated colorectal lesions (with video) Gastrointest Endosc 2024 99 83 9.0E82 10.1016/j.gie.2023.07.025 37481003

[4] Wedi E Fischer A Hochberger J Multicenter evaluation of first-line endoscopic treatment with the OTSC in acute non-variceal upper gastrointestinal bleeding and comparison with the Rockall cohort: the FLETRock study Surg Endoscopy 2017 32 307 314 10.1007/s00464-017-5678-7 28656336

